# Imipenem/cilastatin sodium (IPM/CS) as an embolic agent for transcatheter arterial embolisation: a preliminary clinical study of gastrointestinal bleeding from neoplasms

**DOI:** 10.1186/2193-1801-2-344

**Published:** 2013-07-26

**Authors:** Reiko Woodhams, Hiroshi Nishimaki, Go Ogasawara, Kaoru Fujii, Takuro Yamane, Kenichiro Ishida, Fumie Kashimi, Keiji Matsunaga, Masakazu Takigawa

**Affiliations:** Department of Diagnostic Radiology, Kitasato University School of Medicine, 1-15-1, Kitasato, Sagamihara, Minami-ku, Kanagawa, 252-0374 Japan; Department of Cardiovascular Surgery, St. Marianna University School of Medicine, 2-16-1, Miyamae-ku, Kawasaki, Kanagawa, 216-8511 Japan; Department of Critical care and Emergency Medicine, Kitasato University School of Medicine, 1-15-1, Kitasato, Sagamihara, Minami-ku, Kanagawa, 252-0374 Japan; Department of Radiology, National Hospital Organization, Sagamihara National Hospital, 18-1, Sakuradai, Minami-ku, Sagamihara, Kanawaga, 252-0392 Japan

**Keywords:** Bleeding, Neoplasm, Embolisation, IPM/CS

## Abstract

**Purpose:**

To evaluate the feasibility and usefulness of imipenem/cilastatin sodium (IPM/CS) as an embolic agent for intestinal bleeding from neoplasms.

**Materials and methods:**

Seven patients who underwent 11 transarterial embolisations (TAEs) using IPM/CS as an embolic material for duodenal or small/large intestinal tumour bleeding from January 2004 to December 2011 were retrospectively evaluated. A mixture of IPM/CS and contrast medium was introduced through the microcatheter positioned at the feeding artery to the tumour until extravasation disappeared or stasis of blood flow to the tumour staining was observed.

**Results:**

Haemostasis was obtained in all patients. Therefore, the technical success rate was 100%. Rebleeding was observed in four patients. All of them underwent repeat TAE using IPM/CS, and haemostasis was obtained successfully. No complication was identified following laboratory and clinical examinations. No haemorrhagic death occurred. Haemorrhagic parameters, including blood haemoglobin and the amount of blood transfusion, improved after TAE.

**Conclusion:**

The safety, feasibility, and effectiveness of TAE using IPM/CS as an embolic material for intestinal bleeding from neoplasms were suggested by this study. The mild embolic effect of IPM/CS may be adequate for oozing from tumours. Although rebleeding may occur after embolotherapy using IPM/CS, repeat embolisation is effective as treatment for rebleeding.

## Introduction

Control of tumour bleeding is a compelling need for physicians. Anticancer therapy, including resection, chemotherapy, and radiation, can be a solution for bleeding from neoplasms. Transcatheter arterial embolisation (TAE) may be selected as rescue therapy for patients in whom haemostasis is not achievable with these treatments.

Various embolic materials including metallic coils, gelatin sponges, *n*-butyl 2-cyanoac\rylate, polyvinyl alcohol (PVA), and microspheres have been used for TAE for intestinal bleeding (Kramer et al. 2000; Defreyne et al. 2001). When the bleeding origin vessel is well visualised and superselectively accessible, coil embolisation will be selected (Defreyne et al. 2001; Gordon et al. 1997; Horiguchi et al. 2003; Ledermann et al. 1999). However, it may be difficult to identify culprit vessels of tumour bleeding on angiography because tumour bleeding is likely to be attributed to fine vessels (Chuang et al. 1979). Additionally, tumour bleeding is sometimes intermittent, and extravasation may not be observed at the time of angiography. In these situations, superselective embolisation using coils will not be feasible, and use of gelatin sponges or PVA may be considered. However, embolisation with these agents may cause ischaemic complications of neighbouring intact structures, particularly for small and large intestinal bleeding, because of relatively poor collateral blood supply (Defreyne et al. 2001; Guy et al. 1992; Rosenkrantz et al. 1982). Pancreatitis and duodenal stricture have been reported as complications after superselective TAE of the gastroduodenal artery, mostly using glue and a gelatin sponge (Lang 1992; Loffroy & Guiu 2009).

In 1999, two Japanese studies reported introduction of imipenem/cilastatin sodium (IPM/CS) as an embolic agent for chemoembolisation in an animal model, in which IPM/CS particles in a mixture with iodine contrast material were revealed as small in size, around 10 to 70 μm, and demonstrated a transient embolic effect (Aihara 1999a; Aihara 1999b). Based on these characteristics of IPM/CS, we assumed the usefulness of IPM/CS as an embolic agent for intestinal bleeding from neoplasms. In clinical practice, we performed embolotherapy using IPM/CS for patients who needed haemostasis for bleeding from neoplasms, and who were poor candidates for other therapeutic options such as endoscopic, surgical haemostasis and embolotherapy with other embolic materials because of the possibility of ischemic complication. Arterial introduction of IPM/CS as a prophylactic antibiotic for acute pancreatitis has been performed (Buchler et al. 2000), indicating the safety of arterial introduction of IPM/CS.

The purpose of the present study was to evaluate the feasibility and usefulness of IPM/CS as an embolic material for intestinal bleeding from neoplasms.

## Materials and methods

### Subjects

The Ethics Committee of Kitasato University Hospital approved the present study

Indications for TAE were decided at angiography based on active extravasation or tumour staining of the known tumour. When TAE was indicated, a microcatheter was advanced into the artery whose blood supply covered the area of extravasation as well as the area of tumour staining.

Second, patients who bled from the duodenum or small/ large intestine were chosen as candidates for use of IPM/CS. Subsequently, the indication for IPM/CS was determined when one of the following findings was observed on the angiogram: 1) the bleeding artery was too small to place the metallic coil; 2) extravasation was observed, but the specific bleeding origin artery was hard to detect; or 3) tumour staining, vascular encasement, or vascular proliferation relating to the neoplasm was observed, although no extravasation was observed. These criteria were defined based on speculation that the size of the IPM/CS particle, mainly distributed from 10 to 70 μm (Aihara 1999a; Aihara 1999b), may be adequate to embolise bleeding from small vessels. Patients who showed unstable vital signs or were in shock were excluded from the indication of IPM/CS.

Seven patients (five men and two women) underwent 11 TAE procedures using IPM/CS for duodenal or small/large intestinal bleeding from neoplasms from January 2004 to December 2011. Their ages ranged from 43 to 73 years (mean age: 59.2 ± 11.0 years). Four patients had repeat TAE using IPM/CS.

All patients owned the neoplasm in the large or small intestine at the time of intestinal bleeding”. The median time from the onset of bleeding to angiography was 6 days, ranging from 0 to 20 days. The origins of bleeding included invasion of pancreatic cancer to the duodenum (n = 2), metastasis of renal cell carcinoma to the pancreas invading to the duodenum (n = 1), invasion of cervical cancer to the rectum (n = 1), colon cancer (n = 1), and malignant lymphoma of the small intestine (n = 2). No patient was a candidate for operative haemostasis due to progression of the neoplasm (n = 6) or pulmonary thromboembolism (n = 1) at the time of bleeding. Four of seven patients had endoscopy before angiography to attain haemostasis. Two of these four patients had rebleeding after endoscopic thermocoagulation. In the other two patients, the endoscope could not reach the bleeding sites because of narrowing of the intestinal lumens caused by the tumours.

### Angiography

First, a 5 F introducer was placed in the right or left femoral artery. Angiography of the celiac artery, superior mesenteric artery (SMA), or inferior mesenteric artery (IMA) was performed using a 5 F cobra or shepherd-type catheter by injecting 20 to 25 ml of contrast medium at a rate of 4.0 to 5.0 ml/sec for the SMA and celiac artery and 10 ml to 15 ml at a rate of 2.0 to 2.5 ml/sec for the IMA.

### Preparation and deployment of the IPM/CS mixture

A mixture of 0.5 g IPM/CS and 5 ml of contrast medium was prepared for use as an embolic agent. Five millilitres of contrast medium was put in a vial containing 0.5 g IMP/CS and the mixture was stirred. The mixture was then drawn into a 5-ml syringe and pumped gently with a 2.5-ml syringe connected to the 5-ml syringe through a three-way stopcock. This mixture was injected through a microcatheter placed at the artery whose blood supply covered the tumour staining until the extravasation disappeared or stasis of blood flow to the tumour staining was observed.

### Analysis

The TAE procedure was deemed technically successful when disappearance of extravasation or tumour staining was attained at the time of the procedure.

All patients were carefully followed up with clinical and laboratory examinations to check for rebleeding and complications from embolisation for at least 1 week at the hospital. Rebleeding was deemed positive when melena, haematochezia, or haematemesis was observed. Haematologic parameters, including the total number of transfused units of packed red blood cells and averaged haemoglobin for 7 days, were compared before and after TAE to assess the effect of TAE.

Each patient was assessed for peritoneal signs and symptoms such as tenderness, nausea, diarrhoea, fever, and marked change in bowel sounds after embolisation to determine the occurrence of ischaemic injury every day for at least 1 week. Laboratory ischaemic evidence was evaluated by assessing the increase in white blood cell count (WBC), creatine phosphokinase (CPK), and lactate dehydrogenase (LDH) by comparison of averaged data of the patients for 7 days before and after TAE. In evaluating the volume of blood transfusion, averaged haemoglobin, WBC, CPK, and LDH for the two patients who had repeat TAE within 24 hours after the first TAE (patients 2 and 6), the data until the first TAE were deemed the data before TAE, and data after the second TAE were deemed the data after TAE. One patient underwent upper gastrointestinal endoscopy after TAE to explore haemostasis and to check for complications (patient 3). Two patients were transferred to another hospital to continue treatment for cancer after 44 and 339 days, respectively. The period of clinical follow-up to check for rebleeding ranged from 37 to 339 days after TAE (median, 163 days).

### Statistics

The Wilcoxon signed-rank test was used to compare laboratory data and blood transfusion volume before and after TAE using JMP 7.0 software (2007 SAS institute, Inc., Cary, NC).

## Results

**Figure 1:**
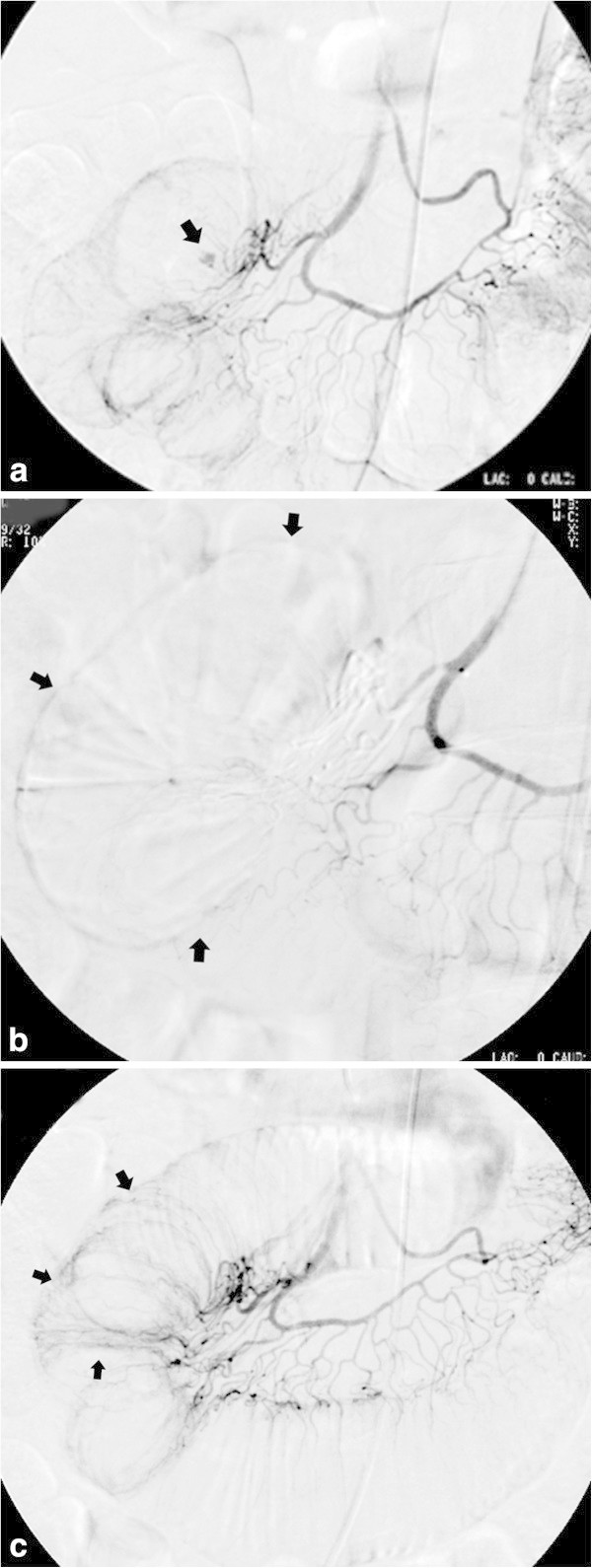
**A 59-year-old man diagnosed with malignant lymphoma at the jejunum had melena for 2 days (patient 2).** Angiography from the jejunal artery **(a)** showed extravasation of the contrast medium (arrow). TAE with 0.2 g IPM/CS was performed through a microcatheter placed at a site proximal to the arterial arcade of the jejunal artery. Angiography post embolisation **(b)** showed disappearance of extravasation and devascularisation around the extravasation point (arrows). On the following day of the first TAE, angiography was repeated because of haematemesis and melena, which suggested rebleeding. Recanalisation of the jejunal segment was observed without extravasation **(c)**. Embolisation using 0.6 g IPM/CS was performed from the same site of the jejunal artery as the first TAE. Haemostasis was successfully obtained and no clinical or laboratory sign suggesting rebleeding was observed after the second TAE. The patient died at 163 days after TAE because of the progression of malignant lymphoma.

A summary of the clinical course of the seven patients (11 procedures) is shown in Table 1. Angiography revealed tumour staining in all patients (Figure 1). Extravasation was observed in three of the eleven procedures. However, bleeding origin vessels accessible by microcatheter were not found in any of the three procedures (Figure 1). All 11 procedures showed encased arteries, proliferation of blood vessels, and prolonged staining with contrast enhancement at locations of the known tumours, which were strongly assumed to be origins of bleeding.

**Table 1:** **Summary of clinical course**

Pt. no	Age	Sex	Disease	Outcome	Proc no.	Extravasation	Embolized artery	IPM/CS (g)	Rebleeding (day)
1	53	M	Pancreatic cancer	Died at day 247	1	+	PA	0.5	-
2	59	M	Malignat lymphoma	Died at day 163	2	+	JA	2.2	0
					3		JA	0.6	-
3	68	M	Pancreatic lymphoma	Died at day 48	4		IA	0.5	21
					5		IA	1.0	1
4	73	M	Pancreatic cancer	Transferred at day 44	6		PA	1.5	10
					7		PA	1.4	-
5	64	M	Metastasis to the pancreas	Died at day 224	8		PA	0.9	-
6	46	F	Invasion of cervical cancer to the rectum	Transferred at day 339	9 10		IRA	0.4	0
							SRA	0.4	-
7	43	F	Colon cancer	Died at day 37	11	+	LCA		-

Pt. no., patient number; Proc. no., procedure number; PA, pancreatic arcade; JA, jejunal artery; IA, ileal artery; RCC, renal cell carcinoma; IRA, inferior rectum artery; SRA, superior rectum artery; LCA; left colic artery.

The positions of the microcatheter during embolisation were at the main trunk of the pancreatic arcade (4 procedures), at a site proximal to the arterial arcade of the jejunal artery (2 procedures), at a site proximal to the arterial arcade of the ileal artery (2 procedures), at a site proximal to the marginal artery of the left colic artery (1 procedure), at the main trunk of superior rectal artery (1 procedure), and at the main trunk of the inferior rectal artery (1 procedure). Disappearance of extravasation or blood flow to the tumour during the procedure was obtained in all procedures. Thus, the technical success rate was 100%. No complication was observed relating to the TAE technique. The median amount of IPM/CS used for TAE was 0.6 g (range: 0.2–1.5 g).

Bleeding recurred for five procedures (45%) in four patients at 0, 0, 1, 10, and 21 days after TAE. TAE was repeated after four of the five procedures and was successful in all procedures. During all these repeat TAE procedures, recanalisation of the arteries that were embolised at the first TAE was observed (Figure 1c). For the other patient suffering from rebleeding, haemostasis occurred without repeat TAE. No conversion to surgical haemostasis occurred.

The haematologic data are shown in Table 2. Transfusion of packed red blood cells was performed for all patients before TAE. After TAE, the volume of transfusion of packed red blood cells was significantly reduced (*P* = 0.0004). For six procedures, transfusion of blood cells was not required for at least 7 days after TAE. The average haemoglobin in 7 days increased after TAE, but no statistically significant difference was found (8.1 g/dl and 9.2 g/dl before and after TAE, respectively; *P* = 0.05).

**Table 2:** **Haematologic parameters**

Pt. no.	Proc. no.	Averaged Hb (g/dl)		Units of blood transfusion	
		Before TAE	After TAE	Before TAE	After TAE
1	1	7.3	9.1	26	10
2	2	11.3		24	-
	3	10.0			0
3	4	9.0	10.5	6	0
	5	6.5	8.5	22	8
4	6	8.0	8.2	8	0
	7	7.2	8.1	8	0
5	8	7.2	10.7	10	0
6	9	7.4		12	-
	10		9.1		10
7	11	9.4	8.7	4	0

Pt. no., patient number; Proc., no., procedure number; Hb, haemoglobin level.

No clinical sign or symptom of ischaemia after TAE was observed. The mean WBC, LDH, and CPK before and after TAE were as follows; WBC (μl): 8637 ± 3243, 10212 ± 3490, LDH (IU/l): 275 ± 250, 285 ± 244, CPK (IU/l): 33 ± 20, 38 ± 30. No significant difference in WBC, LDH, or CPK was found before and after TAE . In the patient who had two TAE procedures and endoscopies after each TAE (patient 3), no sign of rebleeding or ischaemic change was noted.

No death directly relevant to tumour bleeding or embolisation occurred. Five patients died because of progression of the original diseases 37 to 247 days (median: 163 days) after TAE.

## Discussion

We experienced no adverse event related to arterial introduction of IPM/CS, suggesting the safety of IPM/CS as an embolic material. The 100% technical success rate indicated the feasibility of TAE using IPM/CS for bleeding from neoplasms. The effectiveness of TAE using IPM/CS was suggested by stabilisation of blood haemoglobin after TAE with a decreased volume of blood transfusion. Lack of haemorrhagic death further supports the effectiveness of TAE. Our preliminary experiences indicated that TAE using IPM/CS may contribute to reduce the blood transfusion.

Proximal embolisation using a gelatin sponge and metallic coil is not recommended because of the possibility of ischaemic complications (Gordon et al. 1997). In our series, although IPM/CS was introduced from the proximal site of the artery to cover the tumour staining, no major ischaemic complication was suggested based on physical examinations and laboratory data. Although some previous studies have shown successful embolisation using PVA introduced from the proximal site for gastrointestinal bleeding (Defreyne et al. 2001; Loffroy et al. 2010), the risk of causing an ischaemic event has been demonstrated in another report (Guy et al. 1992). Recently, Okuno et al. presented an evaluation of feasibility and effects of TAE using IPM/CS as an embolic agent to treat tendinopathy and enthesopathy (Okuno et al. 2013). In this study, IPM/CS with the same concentration as our study was introduced from relatively proximal point of the branches feeding the target site. Nevertheless, there was no adverse event relating to IPM/CS embolization. Although, the embolized organs are different, Okuno’s study may support the safety of IPM/CS as an embolic agent. The absence of ischaemic complication after proximal IPM/CS embolisation in our study was considered due to the transient embolic effect of IPM/CS, which was demonstrated in our study based on the findings of recanalisation on repeat TAE.

IPM/CS is an antibiotic agent, and it is slightly soluble in water. Therefore, IPM/CS ought to dissolve over time. The transient embolic effect of IPM/CS maybe responsible for rebleeding. Rebleeding occurred in four patients in the present study. The rebleeding ratio in our study was higher than that in previous reports using PVA, glue, a gelatin sponge, and a metallic coil for gastrointestinal bleeding (Defreyne et al. 2001; Guy et al. 1992). However, all repeat TAE procedures after rebleeding were successful in our series. Because safety should be a compelling priority in the treatment of chronic tumour bleeding, transient embolisation with IPM/CS may be one of the viable therapeutic options if repetition is acceptable. Additionally, because of the small particle size of IPM/CS, it would be difficult to stop massive bleeding using IPM/CS, and its application should be restricted to the mild, oozing type of bleeding.

A major limitation of our study is the small number of patients studied.

Another limitation is no proof of macroscopic hemostasis because the patients did not receive operations or endoscopy. The superiority of IPM/CS over other embolic materials as an embolic material for bleeding from neoplasms was not evaluated in this study because of the nature of retrospective study. A further study to compare between IPM/CS and other embolic materials is needed.

In conclusion, our preliminary observations suggest the feasibility of TAE using IPM/CS as an embolic material for oozing from the tumour at the duodenum as well as the small and large intestines. The favourable result of the present preliminary, retrospective study warrants a prospective investigation in a larger population to determine the safety and effectiveness of TAE using IPM/CS.
